# Oral administration of γ-aminobutyric acid affects heat production in a hot environment in resting humans

**DOI:** 10.1186/1880-6805-31-3

**Published:** 2012-02-29

**Authors:** Taiki Miyazawa, Takashi Kawabata, Kazunobu Okazaki, Takashi Suzuki, Daiki Imai, Takeshi Hamamoto, Shinya Matsumura, Toshiaki Miyagawa

**Affiliations:** 1 Department of Environmental Physiology for Exercise, Graduate School of Medicine, Osaka City University, 3-3-138 Sugimoto, Sumiyoshi-ku, Osaka 558-8585, Japan; 2 Research Center for Urban Health and Sports, Osaka City University, 3-3-138 Sugimoto, Sumiyoshi-ku, Osaka 558-8585, Japan; 3 Department of Biomedical Engineering, National Cardiovascular Center Research Institute, 5-7-1 Fujishirodai, Suita, Japan; 4 Food R&D Center, Japan Tobacco Inc, 5-14 Haneda Asahi-cho, Ota-ku, Tokyo 144-0042, Japan; 5 Department of Sport Science and Medical Science, Graduate School of Sport and Exercise Science, Osaka University of Health and Sport Sciences, 1-1 Asashirodai, Kumatori-cho, Sennan-gun, Osaka 590-0496, Japan

**Keywords:** sweat rate, rest, temperature regulation, esophageal temperature

## Abstract

**Background:**

Central administration of γ-amino butyric acid (GABA) induces lower body temperature in animals in hot ambient air. However, it is still unknown whether oral GABA administration affects temperature regulation at rest in a hot environment in humans. Therefore, in the present study, we specifically hypothesized that systemic administration of GABA in humans would induce hypothermia in a hot environment and that this response would be observed in association with decreased heat production.

**Methods:**

Eight male participants drank a 200-ml sports drink with 1 g of GABA (trial G) or without GABA (trial C), then rested for 30 minutes in a sitting position in a hot environment (ambient air temperature 33°C, relative humidity 50%).

**Results:**

We found that changes in esophageal temperature from before drinking the sports drink were lower in trial G than in trial C (-0.046 ± 0.079°C vs 0.001 ± 0.063°C; *P *< 0.05), with lower heat production calculated by oxygen consumption (41 ± 5 W/m^2 ^vs 47 ± 8 W/m^2^; *P *< 0.05).

**Conclusions:**

In this study, we have demonstrated that a single oral administration of GABA induced a larger decrease in body core temperature compared to a control condition during rest in a hot environment and that this response was concomitant with a decrease in total heat production.

## Background

γ-aminobutyric acid (GABA) is an amino acid that is widely distributed throughout the central nervous system (CNS) and is the most important depressive neurotransmitter [1]. GABA has important roles concerning temperature regulation in the hypothalamus. In experimental animals, it has been reported that central pharmacological stimulation of GABA in the dorsomedial hypothalamus (DMH) and the posterior hypothalamus (PH) inhibits heat production [2,3]. On the contrary, it has been reported that central pharmacological stimulation of GABA in the preoptic (PO) area and anterior hypothalamus (AH) increases heat production [4,5]. On the basis of these reports, it is assumed that central GABA stimulation to the hypothalamic region has some practical effect on temperature regulation.

Few studies have examined the influence of systemic GABA stimulation on temperature regulation in either experimental animals or humans because the blood-brain barrier is impermeable to GABA [6] and it has long been thought that systemic administration of GABA cannot affect GABA's availability in the CNS [7,8]. However, it has been suggested that GABA could access certain areas of the brain that lack the blood-brain barrier [9-11], such as the hypothalamus. Therefore, it is expected that systemically administered GABA affects GABA's availability in the hypothalamus and may have effects on the responses of temperature regulation in humans, as reported with regard to central pharmacological stimulation in experimental animals.

On the basis of a previous study conducted in our laboratory, we have reported that oral GABA administration might induce lower core temperature during rest under a hot environment [12]. However, because our previous study was focused on the effect of oral GABA administration during exercise, we derived insufficient data to clarify the effect of oral GABA administration during rest. For example, the elapsed time from GABA administration differed among the subjects because the beginning of the rest period was determined to be the time at which the core temperature returned to the baseline level after sample ingestion. Furthermore, it is thought that subjects were under a certain amount of mental or physical stress because they maintained a semirecumbent position with their feet on riding pedals to be ready for exercise and had a catheter inserted into the left antecubital vein for blood sampling. Such physical and/or mental stresses could affect physiological parameters. Therefore, it is still unknown whether oral GABA administration affects temperature regulation at rest in a hot environment. In the present study, we specifically hypothesized that systemic administration of GABA in humans would induce hypothermia in a hot environment and that this response would be observed in association with decreased heat production. To test these hypotheses in the present study, we compared temperature-regulatory responses in resting humans in a hot environment between two trials: after oral GABA administration and after placebo administration. If we could detect the hypothermic effect of systemic administration of GABA in a hot environment in humans, we expected that oral GABA administration would be used to prevent hyperthermia and the resulting heat disorders that occur during high-temperature conditions, such as during the summer.

## Methods

### Participants

Eight healthy nonsmoking men who usually exercised 3 to 5 days per week were included in this study. Their physical characteristics were age 23.5 ± 3.6 years, height 172.6 ± 3.5 cm, weight 66.9 ± 7.4 kg, body mass index 22.5 ± 2.7 kg/m^2 ^(all means ± SD). Written informed consent was obtained from all participants before participation in this study. The study protocol and the informed consent forms, which conformed to the guidelines of the Declaration of Helsinki, were approved by the Ethics Committee of the Graduate School of Medicine at Osaka City University.

### Experimental design

This study was carried out using a placebo-controlled, double-blind design. The participants randomly performed two trials, a control trial (trial C) and a GABA trial (trial G). The trials were conducted at the same time of day with an interval of at least 3 days between trials to avoid the influence of circadian variation. All experiments were conducted in the morning during the winter season (from January to March). The participants ingested a 200-ml sports drink (placebo) in trial C and a 200-ml sports drink containing 1 g of GABA (GABA drink) in trial G. The dose was the maximal consumption of GABA for a single ingestion (Japan Standard Commodity Classification 872199). Both the placebo and the GABA drink were stored at room temperature controlled at 23°C to 25°C. We checked and confirmed that the temperature of each fluid was 21°C to 23°C before the participants drank it. The sports drink (200 ml) provided 30 kcal of energy, 7.4 g of carbohydrate, 0 g of protein and 0 g of fat.

### Protocols

The participants were requested to refrain from consuming food or drink for at least 12 hours and to avoid caffeinated beverages, alcohol and strenuous physical activity for at least 24 hours before each experiment. Consumption of drinking water was permitted as required. The participants reported to the laboratory at 10:00 AM. They were asked to drink water (200 ml) and then sit for 30 minutes to achieve a normal body fluid balance. They then voided, were weighed in the nude and were provided a pair of shorts. They then swallowed an esophageal thermistor. Subsequently, they entered the climatic chamber (TBR-6W2S2L2M; ESPEC Corp, Osaka, Japan) maintained at an ambient temperature (T_a_) of 33°C and 50% relative humidity. The participants sat for 1 hour on chairs without backrests while measurement devices were positioned. After baseline data correction for 5 minutes and confirmation of stable physiological responses before drinking, the participants ingested the placebo in trial C and the GABA drink in trial G within 1 minute. They were then instructed to rest in a sitting position for 30 minutes. Because we had confirmed that plasma GABA concentration increased after oral administration of GABA, peaked 20 to 40 minutes after ingestion and maintained significantly higher levels than the baseline for at least 1 hour [12], we set the rest period for 30 minutes in this study. During the rest period, an examiner in close proximity to each subject paid close attention to prevent a reduction in the subject's arousal level by checking the subject's heart rate and facial expression. After the rest period, the monitoring instruments were promptly removed, and the participants exited the chamber, wiped themselves down and were weighed again in the nude to estimate total sweat loss.

### Measurements

Esophageal temperature (T_es_) was measured using the esophageal thermistor inserted into a polyethylene tube (LT-ST08-11; Gram Co, Saitama, Japan). The tip of the tube was advanced through the external nares to a distance of one-fourth the participant's standing height. Skin temperature was measured using thermistors (LT-ST08-12; Gram Co) placed on the chest (T_chest_), upper arm (T_arm_), thigh (T_thigh_) and leg (T_leg_) on the left side. Mean skin temperature (T_sk_) was calculated from the body surface area distribution and thermal sensitivity of each skin area using the following formula, which was proposed by Ramanathan [13]:

$${\text{T}}_{\text{sk}}=0.3\left({\text{T}}_{\text{chest}}+\;{\text{T}}_{\text{arm}}\right)+0.2\left({\text{T}}_{\text{thigh}}+\;{\text{T}}_{\text{leg}}\right)$$

T_a _was measured using a thermister (LT-ST08-12; Gram Co) in the climate chamber at the same level of the chest thermister. The precision of LT-ST08-11 and LT-ST08-12 was ± 0.01°C. Heart rate (HR) measured by electrocardiography and blood pressure measured by oscillometry were measured noninvasively every minute (BSM-4103; Nihon-Kohden, Tokyo, Japan). Mean arterial pressure (MAP) was calculated as one-third of the pulse pressure plus the diastolic blood pressure. Skin blood flow was measured on the chest (SkBF_chest_) on the left side by laser Doppler flowmetry (ALF21D; Advance, Tokyo, Japan). Sweat rate was measured on the chest (SR_chest_) on the left side using the ventricular capsule method (SS-100ΙΙ; K and S, Aichi, Japan). The flow rate and duration of air ventilation before starting measurement were 0.3 ml/minute and 10 minutes, respectively. The precision of the SS-100ΙΙ for SR measurements was ± 3% within the range of 0 to 5 mg/cm^2^/minute. For each participant, the probe and capsule were placed on the skin at identical sites in both trials. Data for T_es_, skin temperature, T_a_, SkBF_chest _and SR_chest _were collected at intervals of 1 second using a 16-channel computerized data acquisition system (Intercross-310; Intercross Co, Tokyo, Japan) and stored in data files on a laboratory computer (JPA32301WP; Hewlett-Packard Japan Ltd, Tokyo, Japan). Oxygen consumption (VO_2_) was measured at 20-second intervals using a metabolic gas analyzer system (Vmax Encore 29 System; VIASYS Healthcare, Inc, Yorba Linda, CA, USA). The coefficient of variation for VO_2 _measurement by the Vmax Encore 29 System was 3%. Total sweat loss was estimated by determining the change in dry body weight (BW), which was measured immediately before and after the experiment as (BW_before_) - (BW_after_) + 200 g (weight of drink). There was no significant difference in BW_before _between trial C and trial G (66.96 ± 7.47 kg vs 66.88 ± 7.45 kg; *P *= 0.36).

The absolute values and the changes from baseline of T_es _and T_sk _between 30 minutes of rest were compared between trials. SkBF_chest _values were expressed as percentage changes from the value before drinking (%SkBF_chest _= (SkBF_chest_/before drinking SkBF_chest_) × 100) because absolute values within an individual can vary markedly over the surface of the skin [14]. We averaged the recorded signals in 5-minute time windows.

We calculated the rate of radiant (R), convective (C), conductive (K) and evaporative (E) heat loss (W/m^2^) from the skin surface to the environment using the following equations presented in a previous study [15]:

$$\text{R}={\text{h}}_{\text{r}}\left({\text{T}}_{\text{sk}}-{\text{T}}_{\text{a}}\right),$$

$$\text{C}={\text{h}}_{\text{c}}\left({\text{T}}_{\text{sk}}-{\text{T}}_{\text{a}}\right),$$

$$\text{K}={\text{h}}_{\text{k}}\left({\text{T}}_{\text{sk}}-{\text{T}}_{\text{a}}\right)\text{ and}$$

$$\text{E}={\text{h}}_{\text{e}}\left({\text{P}}_{\text{dp}}-{\text{P}}_{\text{sk}}\right),$$

where h_r_, h_c_, h_k _and h_e _are radiant, convective, conductive and evaporative coefficient of heat transfer, respectively. P_dp _is vapor pressure of room air calculated by T_a _and RH, and P_sk _is saturated vapor pressure at T_sk_. Total heat loss was calculated by summarizing R, C, K and E. Furthermore, we calculated the rate of metabolic energy production as total heat production (M) using the following equation presented previously [15]:

$$\text{M}=\left(0.23\times \text{RER}+0.77\right)\left(5.873\right)\left(\text{V}{\text{O}}_{2}\right)\left(60/\text{Ad}\right),$$

where RER is the respiratory exchange ratio, VO_2 _is oxygen uptake, A_d _is body surface area and 5.873 is oxygen energy equivalent. Heat loss and production were calculated every 1 minute and averaged over time windows of 5 minutes and per 30 minutes.

### Statistical analysis

Two-way (trial-by-time) repeated-measures analysis of variance (ANOVA) was performed to test the effects of GABA administration over time. Subsequent *post hoc *tests to determine significant differences in the various pairwise comparisons were performed using Tukey's *post hoc *test. There was a transient reduction in T_es _after drinking. This response may be due to the sample ingestion and may not be related to GABA effect. Furthermore, this outlier might disturb the interpretation of our statistical analysis. Therefore, the data for T_es _at 5 minutes were excluded from statistical analyses. A paired *t*-test was used to assess differences in heat production, heat loss and total sweat loss between trials. These statistical analyses were computed using SigmaStat version 3.5 software (Systat Software Inc, Chicago, IL, USA). *P *< 0.05 was considered statistically significant. The data are presented as means ± SD unless stated otherwise.

## Results

**Figure 1:**
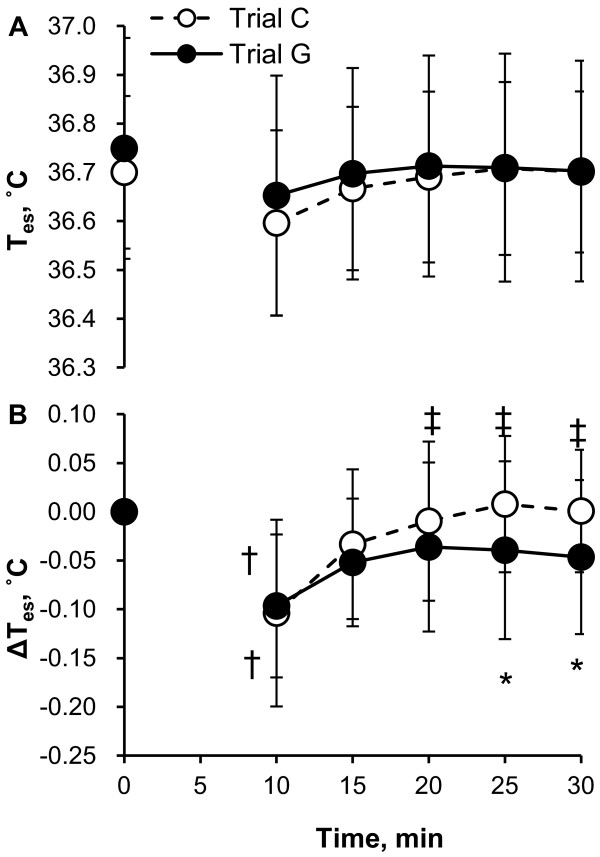
**Time course of esophageal temperature (T_es_) (A, absolute value; B, change from 0-minute value) during 30 minutes of rest in trial C (white) and trial G (black)**. Values are means ± SD for eight participants. *P < 0.05, significant difference between trial C and trial G. ^†,‡^*P *< 0.05, significant difference from 0 minutes and 10 minutes, respectively.

T_a _during 30 minutes of measurement was maintained at 33.01°C to 33.77°C (range of minute average). The absolute values of T_es _and T_sk _at 0 minutes were not significantly different between trial C and trial G (T_es _= 36.70 ± 0.16°C vs 36.75 ± 0.23°C, *P *= 0.40; T_sk _= 34.37 ± 0.55°C vs 34.46 ± 0.47°C, *P *= 0.60). The time course of T_es _is shown in Figure 1 (Figure 1A, absolute value; Figure 1B, change from 0-minute value). There was a transient reduction in T_es _after drinking. This response may be due to the sample ingestion and may not be related to a GABA effect. Furthermore, this outlier might disturb the interpretation of the statistical analysis. Therefore, the data at 5 minutes were excluded from statistical analyses. In trial C, ΔT_es _returned to the same temperature as at 0 minutes after the transient reduction and values at 15 to 30 minutes were not significantly different from those at 0 minutes. However, ΔT_es _at 25 to 30 minutes was significantly higher than at 10 minutes. In trial G, ΔT_es _at 15 to 30 minutes returned to the same temperature as at 0 minutes, and these values were not significantly different from those at 10 minutes. Furthermore, ΔT_es _in trial G was significantly lower than that in trial C at 25 to 30 minutes. There were no significant differences between trials and times in absolute values of T_es_.

**Figure 2:**
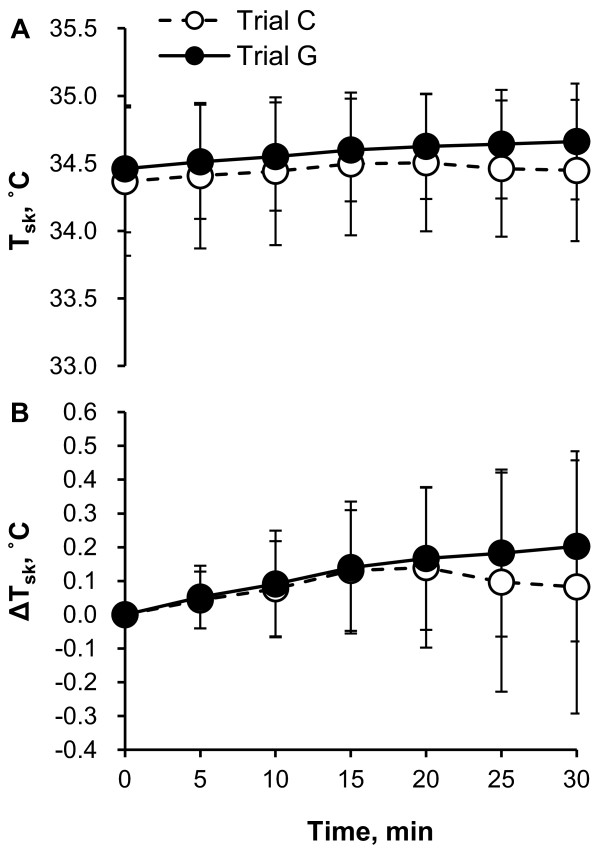
**Time course of mean skin temperature (T_sk_) (A, absolute value; B, change from 0 min value) during 30 min of rest in trial C (white) and trial G (black)**. Values are means ± SD for eight participants.

**Figure 3:**
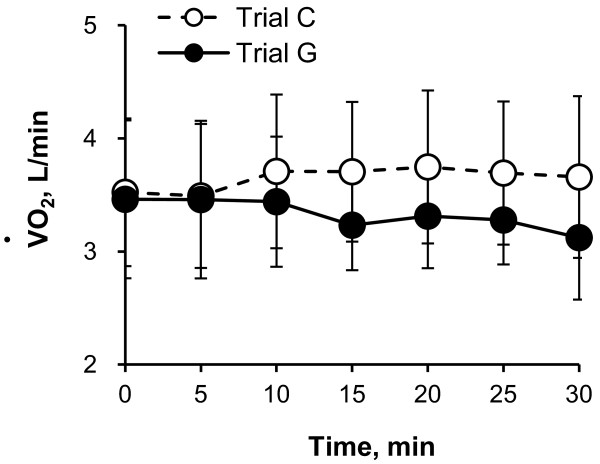
**Time course of oxygen consumption (VO_2_) during 30 minutes of rest in trial C (white) and trial G (black)**. Values are means ± SD for eight participants.

Figure 2 illustrates the time course of T_sk _during 30 minutes of rest (Figure 2A, absolute value; Figure 2B, change from 0-minute value). There was no significant difference in absolute values and changes from 0-minute data for T_sk _between trials and times. The time course of VO_2 _is shown in Figure 3. VO_2 _was slightly lower in trial G than in trial C, although there was no significant difference.

Table 1 shows the values of %SkBF_chest_, SR_chest_, HR and MAP. At 30 minutes, %SkBF_chest_, SR_chest _and HR were unchanged from at 0 minutes throughout the test in both trials and were not significantly different between trials at any time point. MAP at 30 minutes decreased significantly compared with 0 minutes in both trials. Total sweat loss was marginally lower in trial G than in trial C (63 ± 21 g vs 78 ± 30 g; *P *= 0.101).

**Table 1:** Physiological variables^a^

	Trial C		Trial G	
	
Variables	0 minutes	30 minutes	0 minutes	30 minutes
%SkBF_chest _(%)	100	125 ± 49	100	112 ± 19
SR_chest _(mg/cm^2^/minute)	0.14 ± 0.05	0.22 ± 0.13	0.27 ± 0.20	0.23 ± 0.12
HR (bpm)	70 ± 7	71 ± 12	72 ± 11	75 ± 14
MAP (mmHg)	80 ± 9	71 ± 9^b^	78 ± 9	70 ± 10^b^

^a^%SkBF_chest_, percentage change in chest skin blood flow; bpm, beats per minute; SR_chest_, chest sweat rate; HR, heart rate; MAP, mean arterial pressure. ^b^*P *< 0.05, significant difference from levels at 0 minutes. Values are means ± SD for eight participants.

**Figure 4:**
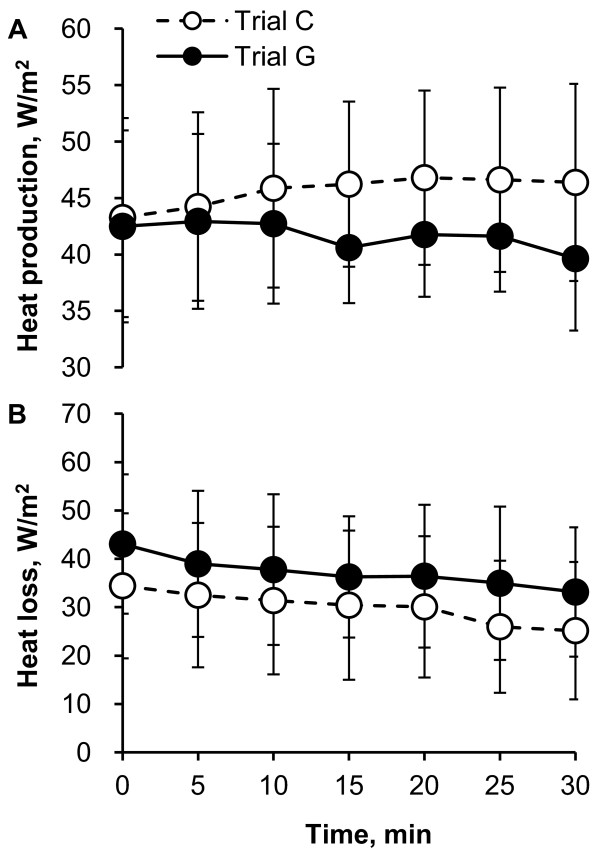
**Time course of heat production (A) and heat loss (B) during 30 minutes of rest in trial C (white) and trial G (black)**. Values are means ± SD for eight participants.

Figure 4 illustrates the time course of heat production (Figure 4A) and heat loss (Figure 4B) during 30 minutes of rest. Heat production was slightly lower in trial G than in trial C, although there was no significant difference. There was no significant difference in heat loss between trials and times.

**Figure 5:**
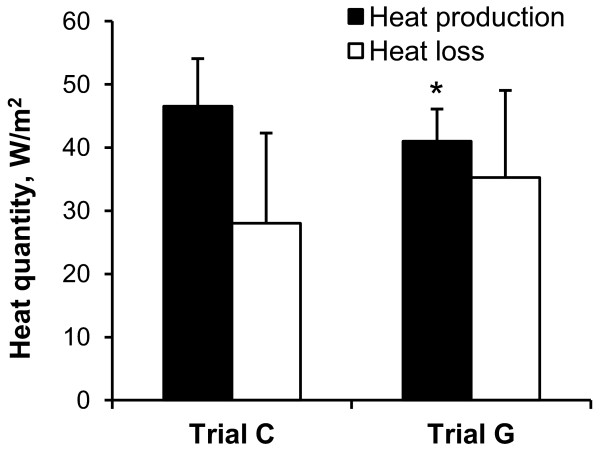
**Mean of heat production (black) and loss (white) during 30 minutes of rest in trial C and trial G**. Values are means ± SD for eight participants. *P < 0.05, significant difference from trial C.

The rate of heat production averaged per 30 minutes in trial G was significantly lower than that of trial C. The rate of heat loss averaged per 30 minutes in trial G was approximately 33% higher than in trial C, but there was no significant difference (Figure 5).

## Discussion

The major findings of the present study are that oral administration of GABA induced a decrease in body core temperature compared to control conditions during rest in a hot environment and that this response was concomitant with a decrease in total heat production.

The most intriguing result of the present study is that the ΔT_es _values in trial G at 25 to 30 minutes were significantly lower than those in trial C, as shown in Figure 1, suggesting that oral administration of GABA induced responses to decrease body core temperature in resting humans in a hot environment. In the conditions of the present study, body core temperature was determined by the balance of heat production with resting metabolism and heat gain from or loss to the environment, such as by radiant, convective and evaporative heat exchange [15]. As shown in Figures 4 and 5, although there were no significant differences in the time course of heat production between trial C and trial G, we observed that heat production averaged per 30 minutes in trial G was lower than that in trial C. Furthermore, although there were no significant differences, the mean data of VO_2 _in trial G were lower than those in trial C from 15 minutes (Figure 3). These data suggest that oral administration of GABA decreased heat production with lowering resting metabolism and therefore decreased the body core temperature. However, it is still unknown how the differences in heat production contribute to the differences in body core temperature. Further studies are needed to more clearly explain the relation between heat production and body core temperature changes.

The responses of ΔT_es _and heat production with oral administration in the present study are experimentally similar to previous observations in animals by showing that central pharmacological stimulation of GABA in the DMH and PH inhibits heat production [2,3]. Neurons in the DMH play key roles in temperature regulation. The activation of DMH elevates core temperature and sympathetic activity to interscapular brown adipose tissue in conscious rats. Microinjection of the GABA_A _receptor agonist muscimol into DMH suppresses increases in sympathetic nerve activity to interscapular brown adipose tissue. In a similar way, the PH can activate an autonomic mechanism controlling heat production in brown adipose tissue. Furthermore, the neural mechanism in the PH mediating this effect is tonically inhibited by GABA. Thus the observations that we present indirectly support the hypothesis that orally administered GABA circulates in the bloodstream and affects GABA concentration and/or the activation of GABA function in the CNS through the region which lacks the blood-brain barrier [9-11] and induces the same effects for the region relating to temperature regulation, such as DMH and PH. However, we found no evidence that orally administered GABA affected central GABA concentration or function in either animals or humans. Additionally, it has been reported in animal studies that when the GABA agonist muscimol perfuses into the PO/AH, heat production and body core temperature are increased in various environmental conditions [4,5]. Therefore, further studies are necessary to elucidate the relationship between the oral administration of GABA and central GABA function in humans as well as in animals.

### Limitations

The present study has some limitations. First, we did not measure plasma GABA concentrations. We confirmed that plasma GABA concentrations increased after oral administration of GABA, peaked 20 to 40 minutes after ingestion and maintained significantly higher levels than the baseline for at least 1 hour [12]. Therefore, to diminish any subject's stress as a result of placing the catheter, we set a rest period of 30 minutes in this study by reference to the previous study. Second, we verified the effect of only one amount of GABA. To clarify the whole picture of the effect of GABA, it is necessary to investigate the effect of other amounts of GABA and reveal the concentration dependency.

## Conclusions

In this study, we have demonstrated that a single oral administration of GABA induced a decrease in body core temperature compared to control condition during rest in a hot environment and that this response was concomitant with a decrease in total heat production.

## Abbreviations

AH: anterior hypothalamus; BW: body weight; C: rate of convective heat loss; CNS: central nervous system; DMH: dorsomedial hypothalamus; E: rate of evaporative heat loss; GABA: γ-amino butyric acid; h_c_: convective coefficient of heat transfer; h_e_: evaporative coefficient of heat transfer; h_k_: conductive coefficient of heat transfer; h_r_: radiant coefficient of heat transfer; HR: heart rate; K: rate of conductive heat loss; M: metabolic energy production; MAP: mean arterial pressure; P_dp_: vapor pressure of room air; PH: posterior hypothalamus; PO: preoptic area; P_sk_: saturated vapor pressure; R: rate of radiant heat loss; RER: respiratory exchange ratio; SkBF_chest_: chest skin blood flow; SR_chest_: chest sweat rate; T_a_: ambient temperature; T_arm_: upper-arm skin temperature; T_chest_: chest skin temperature; T_es_: esophageal temperature; T_leg_: leg skin temperature; T_sk_: mean skin temperature; T_thigh_: thigh skin temperature; VO_2_: oxygen consumption.

## Competing interests

The authors declare that they have no competing interests.

## Authors' contributions

TMiyaz contributed to the conception and design of the experiments. TK, KO and TH drafted the manuscript and revised it critically for intellectual content. TS and DI contributed to data collection, analysis and interpretation. SM contributed to the conception and design of the experiments. TMiyag contributed to the conception and design of the experiments. All authors read and approved the final manuscript.

## References

[B1] Awapara J Occurrence of free γ-aminobutyric acid in brain and its formation from L-glutamic acid Tex Rep Biol Med 1950 8 443 447 14787916

[B2] Dimicco JA Zaretsky DV The dorsomedial hypothalamus: a new player in thermoregulation Am J Physiol Regul Integr Comp Physiol 2007 292 R47 R63 16959861 10.1152/ajpregu.00498.2006

[B3] Amir S Activation of brown adipose tissue thermogenesis by chemical stimulation of the posterior hypothalamus Brain Res 1990 534 303 308 10.1016/0006-8993(90)90145-2 1981483

[B4] Ishiwata T Saito T Hasegawa H Yazawa T Kotani Y Otokawa M Aihara Y Changes of body temperature and thermoregulatory responses of freely moving rats during GABAergic pharmacological stimulation to the preoptic area and anterior hypothalamus in several ambient temperatures Brain Res 2005 1048 32 40 10.1016/j.brainres.2005.04.027 15913569

[B5] Osaka T Cold-induced thermogenesis mediated by GABA in the preoptic area of anesthetized rats Am J Physiol Regul Integr Comp Physiol 2004 287 R306 R313 10.1152/ajpregu.00003.2004 15031132

[B6] Kuriyama K Sze PY Blood-brain barrier to H3-γ-aminobutyric acid in normal and amino oxyacetic acid-treated animals Neuropharmacology 1971 10 103 108 10.1016/0028-3908(71)90013-X 5569303

[B7] Roberts E Baxter CF Metabolic studies of γ-aminobutyric acid Neurology 1958 8 77 78 13541615 10.1212/wnl.8.suppl_1.77

[B8] Tsukada Y Nagata Y Hirano S Active transport of γ-aminobutyric acid in brain cortex slices, with special reference to phosphorus-32 turnover of phospholipids in cytoplasmic particulates Nature 1960 186 474 475 10.1038/186474a0 13839672

[B9] Van Gelder NM Elliott KA Disposition of γ-aminobutyric acid administered to mammals J Neurochem 1958 3 139 143 10.1111/j.1471-4159.1958.tb12620.x 13621235

[B10] Biswas B Carlsson A The effect of intraperitoneally administered GABA on brain monoamine metabolism Naunyn Schmiedebergs Arch Pharmacol 1977 299 47 51 10.1007/BF00508636 904694

[B11] Cavagnini F Invitti C Pinto M Maraschini C Di Landro A Dubini A Marelli A Effect of acute and repeated administration of γ aminobutyric acid (GABA) on growth hormone and prolactin secretion in man Acta Endocrinol (Copenh) 1980 93 149 154 7376786 10.1530/acta.0.0930149

[B12] Miyazawa T Kawabata T Suzuki T Imai D Hamamoto T Yoshikawa T Miyagawa T Effect of oral administration of GABA on temperature regulation in humans during rest and exercise at high ambient temperature Osaka City Med J 2009 55 99 108 20088409

[B13] Ramanathan NL A new weighting system for mean surface temperature of the human body J Appl Physiol 1964 19 531 533 14173555 10.1152/jappl.1964.19.3.531

[B14] Johnson JM Taylor WF Shepherd AP Park MP Laser-Doppler measurement of skin blood flow: comparison with plethysmography J Appl Physiol 1984 56 798 803 10.1063/1.334009 6706783

[B15] Gagge AP Gonzalez RR Fregly MS, Blatteis CM Mechanisms of heat exchange: biophysics and physiology Handbook of Physiology, Section 4. Environmental Physiology 1996 New York: Oxford University Press 45 84

